# APOE interacts with ACE2 inhibiting SARS-CoV-2 cellular entry and inflammation in COVID-19 patients

**DOI:** 10.1038/s41392-022-01118-4

**Published:** 2022-08-01

**Authors:** Hongsheng Zhang, Lin Shao, Zhihao Lin, Quan-Xin Long, Huilong Yuan, Lujian Cai, Guangtong Jiang, Xiaoyi Guo, Renzhi Yang, Zepeng Zhang, Bingchang Zhang, Fan Liu, Zhiyong Li, Qilin Ma, Yun-Wu Zhang, Ai-Long Huang, Zhanxiang Wang, Yingjun Zhao, Huaxi Xu

**Affiliations:** 1 Center for Brain Sciences, the First Affiliated Hospital of Xiamen University, Institute of Neuroscience, Fujian Provincial Key Laboratory of Neurodegenerative Disease and Aging Research, Xiamen University, Xiamen, China; 2 Institute for Brain Science and Disease, Chongqing Medical University, Chongqing, China; 3 School of Medicine, Xiamen University, Xiamen, China; 4 Key Laboratory of Molecular Biology for Infectious Diseases (Ministry of Education), Chongqing Medical University, Chongqing, China

**Keywords:** Infectious diseases, Infectious diseases

## Abstract

Apolipoprotein E (APOE) plays a pivotal role in lipid including cholesterol metabolism. The APOE *ε4* (APOE4) allele is a major genetic risk factor for Alzheimer’s and cardiovascular diseases. Although APOE has recently been associated with increased susceptibility to infections of several viruses, whether and how APOE and its isoforms affect SARS-CoV-2 infection remains unclear. Here, we show that serum concentrations of APOE correlate inversely with levels of cytokine/chemokine in 73 COVID-19 patients. Utilizing multiple protein interaction assays, we demonstrate that APOE3 and APOE4 interact with the SARS-CoV-2 receptor ACE2; and APOE/ACE2 interactions require zinc metallopeptidase domain of ACE2, a key docking site for SARS-CoV-2 Spike protein. In addition, immuno-imaging assays using confocal, super-resolution, and transmission electron microscopies reveal that both APOE3 and APOE4 reduce ACE2/Spike-mediated viral entry into cells. Interestingly, while having a comparable binding affinity to ACE2, APOE4 inhibits viral entry to a lesser extent compared to APOE3, which is likely due to APOE4’s more compact structure and smaller spatial obstacle to compete against Spike binding to ACE2. Furthermore, *APOE ε4* carriers clinically correlate with increased SARS-CoV-2 infection and elevated serum inflammatory factors in 142 COVID-19 patients assessed. Our study suggests a regulatory mechanism underlying SARS-CoV-2 infection through APOE interactions with ACE2, which may explain in part increased COVID-19 infection and disease severity in *APOE ε4* carriers.

## Introduction

COVID-19 (novel coronavirus disease 2019) is a highly infectious disorder caused by SARS-CoV-2 (severe acute respiratory syndrome coronavirus 2). SARS-CoV-2 primarily infects lung epithelial cells through the interaction with its Spike protein to the ACE2 receptor, and then enter the cells via type 2 transmembrane serine proteases (TMPRSS2) mediated cleavage of the Spike protein, which results in the consequent inflammation.^1^ Susceptibility to SARS-CoV-2 and severity of COVID-19 can be affected by the presence of comorbidities (e.g., cardiovascular disease, diabetes), age, immune response, viral load, and genetic variations of individuals.^2–5^ For instance, a higher viral load of SARS-CoV-2 is linked to increased inflammatory markers, and disease severity and mortality in COVID-19 patients.^3^ Several genetic factors have been associated with COVID-19 susceptibility and severity, including *ACE2, TMPRSS2, IL10RB, PLSCR1, ATP11A, MUC1*, and *APOE*.^4–6^ While several of these risk factors are known to be able to regulate cellular entry of SARS-CoV-2 (e.g., *ACE2* and *TMPRSS2*) or immune response (e.g., *IL10RB* and *PLSCR1*), it remains unclear whether and how other factors such as APOE can modulate SARS-CoV-2 infection and the consequently clinical outcomes.

Apolipoprotein E (APOE) is encoded by the *APOE* gene comprising three common allelic variants, *ε2*, *ε3*, and *ε4*. *APOE ε3* is the most represented of all *APOE* genotypes (worldwide average frequency is ~78%); the average frequency of *APOE ε*4 across all races is ~14% (15–20% in Europeans^7^ and 10-19.54% in Chinese population;^8,9^ whereas the frequency of *APOE ε2* is the lowest in all *APOE* alleles (worldwide average frequency is ~8%).^10^ APOE3 and APOE4 are distinguished by a single point substitution at amino acid residue 112: Cys-112 for APOE3 and Arg-112 for APOE4.^11^ The single amino acid substitution results in structural differences between APOE3 and APOE4, which confer differential protein and lipid binding abilities, and consequently different physiopathological roles of these APOE isoforms.^12^ Compelling evidence indicates that *APOE ε4* is a major risk factor for Alzheimer’s and cardiovascular diseases,^13^ where the underlying mechanisms have been extensively investigated.

In this study, we investigated correlations between APOE and immune responses in COVID-19 patients, and determined how APOE and its isoforms regulate SARS-CoV-2 infection in cellular and animal models.

## Results

### APOE levels inversely correlate with SARS-CoV-2-induced inflammation and APOE3 inhibits SARS-CoV-2 pseudo-viral infection

**Fig. 1:**
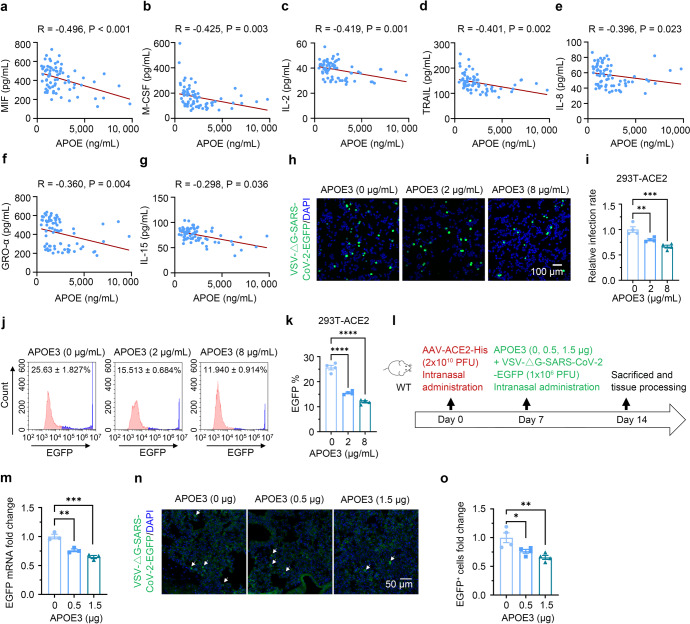
Serum APOE concentrations inversely correlate with inflammation in COVID-19 patients and APOE3 protein dose-dependently inhibits SARS-CoV-2 pseudo-virus infection. **a**–**g** Correlation between APOE and cytokine/chemokine concentrations in serum samples from COVID-19 patients after the adjustment for sex and age. *n* = 73. **h**–**k** Confocal imaging analysis (**h**–**i**) and flow cytometry (**j**–**k**) of VSV-ΔG-SARS-CoV-2-EGFP pseudo-viral load in 293T-ACE2 cells treated with varying concentrations of recombinant APOE3 proteins. *n* = 4 independent experiments. **l**–**o** Effect of APOE3 treatment on the transduction of SARS-CoV2 pseudo-virus in mouse lung expressing human ACE2. **l** Schematic of the study design. **m** qRT-PCR analysis of pseudo-viral EGFP mRNA levels in lung tissues. *n* = 3 mice per group. **n**–**o** Confocal image analysis of VSV-ΔG-SARS-CoV-2-EGFP pseudo-viral load in lung tissues. *n* = 4 mice per group. Data are presented as mean ± S.E.M. One-way ANOVA tests were used to determine statistical significance. *P < 0.05; **P < 0.01; ***P < 0.001; ****P < 0.0001

To investigate the relevance of APOE to SARS-CoV-2-induced inflammatory response, we first examined serum APOE and cytokine/chemokine levels in COVID-19 patients. APOE concentrations did not differ between different sexes, and showed little, if any, correlation with ages of the patients (Supplementary Fig. S1a, b; Supplementary Table S1). We, however, observed a significant inverse correlation between serum concentrations of APOE and levels of GRO (growth regulated protein)-α, M-CSF (macrophage colony-stimulating factor), MIF (macrophage migration inhibitory factor), TRAIL (tumor necrosis factor (TNF)-related apoptosis inducing ligand), IL (interleukin)-2, IL-8, and IL-15, after the adjustment for age and sex (Fig. 1a–g). These results suggest that APOE, especially at high concentrations, may inhibit inflammatory response induced by SARS-CoV-2. As SARS-CoV-2-induced inflammatory response is associated with cellular load of the virus, we initially assessed the effect of APOE3, the most common APOE isoform, on viral infectivity. HEK-293T cells stably expressing human ACE2 (293T-ACE2) were pre-incubated and cultured with recombinant APOE3 protein. We took advantage of VSV-ΔG (G protein deleted) system, which has been widely used to generate pseudotype of SARS-CoV-2 viruses.^14^ Cells were then exposed to VSV-ΔG-SARS-CoV-2-EGFP pseudo-viruses expressing Spike and EGFP, or control VSV-ΔG-EGFP viruses without Spike expression for 24 h. Confocal imaging and flow cytometry analyses showed that APOE3 treatment decreased the amount of SARS-CoV-2 pseudo-viruses in 293T-ACE2 cells in a dose-dependent manner (Fig.1h–k), but had little effect on the transduction of control viruses (Supplementary Fig. S1c, d). APOE3 treatment had no effect on the transduction of either SARS-CoV-2 pseudo-viruses or control VSV-ΔG viruses in naïve 293T cells, which endogenously express low levels of ACE2, ~1200-fold less than that in 293T-ACE2 cells (Supplementary Fig. S1e–i). As nasal administration has been extensively used for delivery of native and pseudo-SARS-CoV-2 viruses, and proteins such as antibodies, into respiratory systems with high efficacies,^15–19^ we employed this approach to investigate the effect of APOE3 on SARS-CoV-2 pseudo-viral transduction in vivo. In a mouse model intranasally transduced with adeno-associated virus expressing human ACE2-His (AAV-ACE2-His), intranasal delivery of APOE3 also dose-dependently inhibited SARS-CoV-2 pseudo-viral infection in the lung (Fig. 1l–o), without affecting ACE2 expression (Supplementary Fig. S1j). These results indicate that APOE protein protects against ACE2/Spike-mediated SARS-CoV-2 like viral infection.

### APOE3 interacts with the ACE2 receptor and reduces ACE2-mediated Spike docking onto the cell surface

**Fig. 2:**
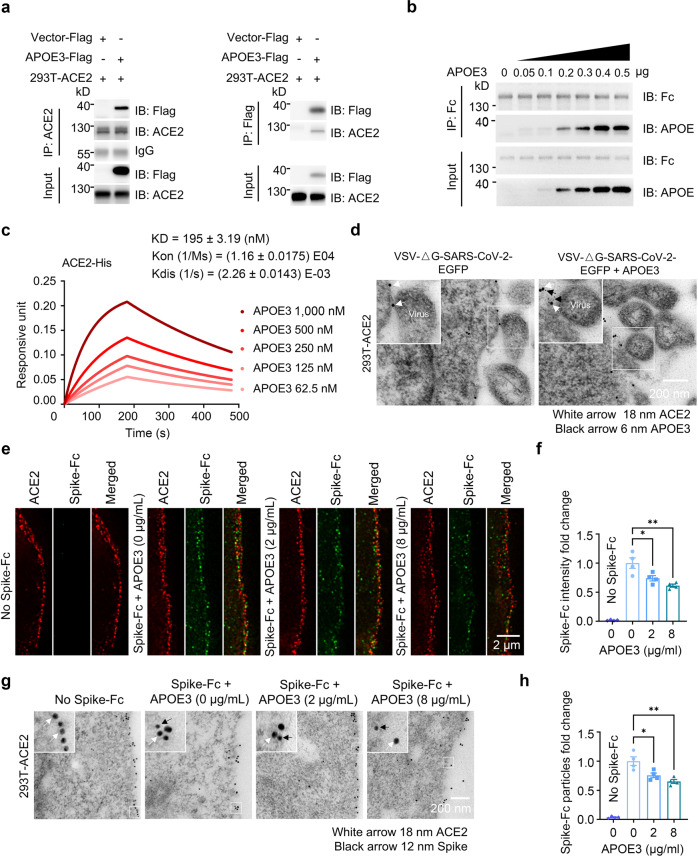
APOE3 interacts with ACE2 and reduces ACE2-mediated Spike docking onto the cell surface. **a** Co-immunoprecipitation between APOE and ACE2 in 293T-ACE2 cells overexpressing APOE3-Flag. **b** Cell-free protein pull-down analysis of ACE2-Fc and recombinant APOE3 protein with indicated amounts. **c** Bio-layer interferometry analysis of immobilized ACE2-Fc proteins bound to APOE3 proteins at the following concentrations: 1000, 500, 250, 125, and 62.5 nM (corresponding to kinetic curves from top to bottom). **d** Immuno-electron microscopic analysis of the localization of ACE2, APOE and the SARS-CoV-2 pseudo-virus in 293T-ACE2 cellular cultures with (right panel) or without APOE treatment (left panel). **e**–**h** Super-resolution (**e**, **f**) and immuno-electron (**g**, **h**) microscopic analyses of the amount of Spike-Fc protein bound to the surface of 293T-ACE2 cells in the presence of recombinant APOE3 proteins at varying concentrations. *n* = 4 independent experiments. Data are presented as mean ± S.E.M. Statistical significances were determined by one-way ANOVA tests. *P < 0.05; **P < 0.01

Since cellular SARS-CoV-2 entry is initiated by docking of the viral Spike protein to the ACE2 receptor,^20^ we tested if APOE can interact with ACE2 and thereby modulate ACE2/Spike protein interactions during viral entry and infection. APOE3 was co-immunoprecipitated with ACE2 in 293T-ACE2 cells overexpressing Flag-tagged APOE3 (Fig. 2a). Cell-free co-precipitation assays showed that recombinant APOE3 protein dose-dependently bound to ACE2-Fc protein, indicating a direct association between APOE3 and ACE2 (Fig. 2b). Biolayer interferometry analyses confirmed the interaction between APOE3 and ACE2 (*K*_D_ = 195 ± 3.19 nM) (Fig. 2c). In addition, we observed a colocalization of APOE3, ACE2 and SARS-CoV-2 pseudo-virus at the surface of 293T-ACE2 cells with recombinant APOE3 treatment, as evidenced by immuno-electron microscopy (Fig. 2d). Moreover, we mapped ACE2 domain that interacts with APOE3, and found that the zinc, but not the D2 domain is required for the ACE2/APOE interaction (Supplementary Fig. S2a, b): specifically, zinc-B and zinc-C regions located near the middle and C-terminus, respectively, but not zinc-A region near the N-terminus of the zinc domain are necessary for the interaction (Supplementary Fig. S2c, d). As the zinc metallopeptidase domain of ACE2 is also required for ACE2/Spike binding,^21,22^ we next determined whether APOE3 can regulate ACE2-mediated Spike protein docking onto the cell surface. In cultured 293T-ACE2 cells, APOE3 treatment significantly decreased the amount of Spike protein bound to the cell surface, as examined by super-resolution and immuno-electron microscopy imaging analyses (Fig. 2e–h). Spike protein that bound to the ACE2 was nearly undetectable in naïve 293T cells, either in the absence or presence of APOE3 treatment (Supplementary Fig. S2e), likely due to the extremely low levels of endogenous ACE2 in 293T cells (Supplementary Fig. S1i). These results demonstrate that APOE3 interacts with ACE2 and this interaction inhibits the docking of Spike protein to ACE2 receptor at the cell surface.

### APOE4 also inhibits cellular entry of SARS-CoV-2 pseudo-viruses by hindering ACE2/Spike interactions and Spike docking onto the cell surface, but to a lesser extent compared to APOE3

**Fig. 3:**
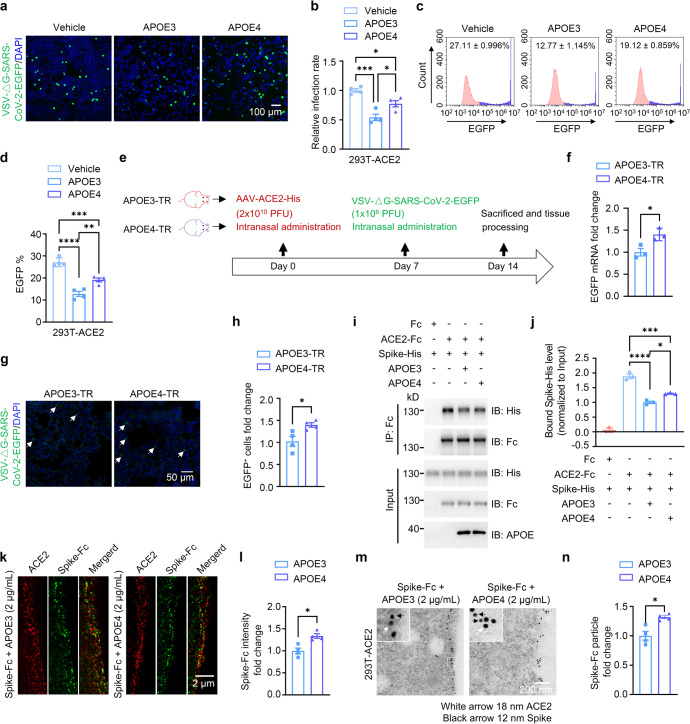
APOE4 features attenuated inhibitory effect on cellular entry of SARS-CoV-2 pseudo-virus, Spike/ACE2 interaction and binding of Spike to the cell surface compared to APOE3. **a**–**d** Confocal imaging (**a**, **b**) and flow cytometry (**c**, **d**) analyses of VSV-ΔG-SARS-CoV-2-EGFP pseudo-viral load in 293T-ACE2 cells in the absence (Vehicle) or the presence of recombinant APOE3 or APOE4 proteins. n = 4 independent experiments. **e**–**h** Assessment of SARS-CoV-2 pseudo-viral load in APOE3-TR or APOE4-TR mouse lung expressing human ACE2. **e** Flow chart of study design. **f** qRT-PCR analysis of pseudo-viral EGFP mRNA levels in APOE3-TR or APOE4-TR mouse lung tissues transduced with AAV-ACE2. *n* = 3 mice per group. **g**, **h** Confocal imaging analysis of VSV-ΔG-SARS-CoV-2-EGFP pseudo-viral load in lung tissues. *n* = 4 mice per group. **i**–**j** Protein pull-down analysis of Spike-His/ACE2-Fc interactions in the presence of recombinant APOE3 or APOE4 proteins. **k**–**n** Super-resolution (**k**, **l**) and immuno-electron (**m**, **n**) microscopic analyses of surface-bound Spike-Fc proteins in 293T-ACE2 cells in the presence of recombinant APOE3 or APOE4 proteins. *n* = 4 independent experiments. Data are presented as mean ± S.E.M. Statistical significances were assessed by unpaired, two-sided Mann–Whitney *U*-tests. *P < 0.05; **P < 0.01; ***P < 0.001; ****P < 0.0001

We next compared effects of APOE polymorphic isoforms on viral infectivity. Analyses by confocal fluorescence imaging and flow cytometry showed that treatment with recombinant APOE4 proteins dose-dependently attenuated the amount of SARS-CoV-2 pseudo-viruses in 293T-ACE2 cells (Supplementary Fig. S3a–d). However, recombinant APOE4 protein treatment showed less inhibitory effect on the pseudo-viral transduction in both 293T-ACE2 and lung epithelial Calu-3 cells, when compared to APOE3 treatment (Fig. 3a–d and Supplementary Fig. S3e). Similarly, treatment of 293T-ACE2 cells with conditioned medium (CM) of rodent primary astrocyte cultures derived from human APOE4 target-replacement mice (APOE4-TR) also reduced intracellular levels of SARS-CoV-2 pseudo-viruses to a lesser extent, when compared to the treatment with CM of astrocyte cultures from human APOE3 target-replacement mice (APOE3-TR) (Supplementary Fig. S3f, g). We then compared effects of APOE polymorphic isoforms on viral infectivity in vivo. Intranasal delivery of AAV-ACE2-His followed by SARS-CoV-2-EGFP pseudo-virus transduction resulted in more accumulation of SARS-CoV-2 pseudo-viruses in the lung of APOE4-TR mice than in that of APOE3-TR mice (Fig. 3e–h). The exogenous human ACE2 expression in lung tissues was nevertheless similar between APOE3-TR and APOE4-TR mice (Supplementary Fig. S3h).

Next, we observed that when 293T-ACE2 cells were pre-incubated with SARS-CoV-2 pseudo-viruses to allow the entry of the viruses for 24 h, subsequent incubation with APOE3 and APOE4 without continuous presence of pseudo-virus yielded no difference in viral expression inside the cells (Supplementary Fig. S3i, j), suggesting that both APOE3 and APOE4 impede cellular entry of SARS-CoV-2 pseudo-viruses without affecting their intracellular expression/replication or propagation. Importantly, we found that although APOE3 and APOE4 comparably bound to ACE2 in co-precipitation (Supplementary Fig. S3k, l) and BLI assays (APOE3 and ACE2 binding affinity: *K*_D_ = 195 ± 3.19 nM, APOE4 and ACE2 binding affinity: *K*_D_ = 190 ± 2.23 nM) (Fig. 2c and Supplementary Fig. S3m), APOE4 reduced interactions between ACE2 and Spike and cell surface-bound Spike protein amount in 293T-ACE2 cells to a lesser extent when compared to APOE3, as measured by co-precipitation (pull-down), immunofluorescent colocalization and immuno-electron microscopy analyses (Fig. 3i–n). Together, these results clearly demonstrate that while APOE4 and APOE3 have comparable binding affinity to ACE2, APOE4 has a much less efficiency than APOE3 in inhibiting ACE2/Spike binding, ACE2-mediated Spike docking onto the cell surface, and the subsequently cellular entry of SARS-CoV-2 pseudo-virus (also see Supplementary Fig. S4b and Discussion).

### APOE *ε4* carriers exhibit increased susceptibility to SARS-CoV-2 and increased serum inflammatory factors in COVID-19 patients

**Fig. 4:**
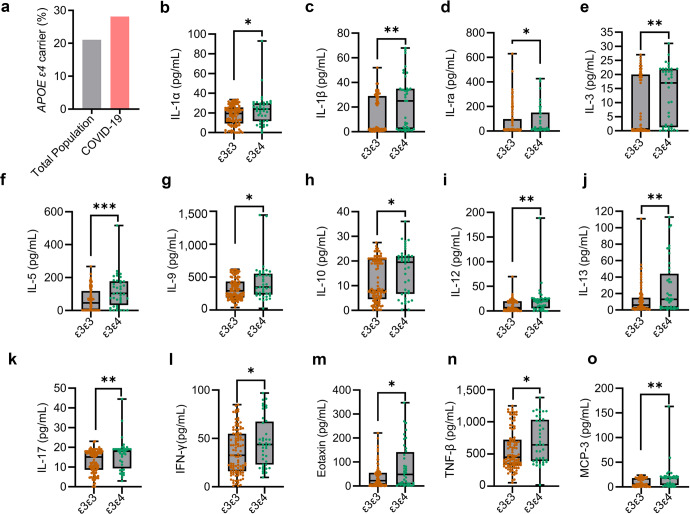
APOE4 is associated with increased COVID-19 incidence and serum indicators of inflammation. **a** The percentage of APOE ε3ε4 carriers in healthy populations (gray) and COVID-19 patient (pink) cohorts based on Jia et al. and our results combined.^8^ **b**–**o** Serum samples from a Chinese cohort of COVID-19 patients with APOE ε3/ε3 (*n* = 102) and ε3/ε4 (*n* = 40) genotypes were collected within the acute phase during hospitalization, and concentrations of cytokines and chemokines in serum samples were determined by ELISA. Box plots represent median, first and third quartiles; Whiskers represent 1.5x the IQR (interquartile range) above and below the box. Data are presented as mean ± S.E.M. Unpaired, two-sided Mann–Whitney *U*-tests were used to evaluate statistical significance. *P < 0.05; **P < 0.01; ***P < 0.001

As APOE3 and APOE4 differentially affect SARS-CoV-2 infectivity, we investigated the association of *APOE* genotype with COVID-19 incidence. *APOE* genotyping by Sanger sequencing and real-time PCR showed that 71.83% subjects are *APOE ε3*/*ε3* (the *ε3* carriers), and 28.17% subjects are *APOE* ε*3*/ε*4* or *ε4*/ε*4* (the *ε4* carriers) in a Chinese cohort of 142 COVID-19 patients excluding APOE *ε2* carriers (Fig. 4a and Supplementary Table S2). Given that the frequency of *ε4* carriers is 21.10% in Chinese population without dementia,^8^ our study revealed an increased infection rate by 33.51% (28.17 vs. 21.10%) for *ε4* carriers (Fig. 4a). In addition, our results corroborate with a previous report that *APOE ε4* genotype correlates with an increased incidence of COVID-19.^5^ Since COVID-19 infectivity and severity can correlate with enhanced inflammatory response,^23^ we examined serum cytokine/chemokine levels in COVID-19 patients. We observed enhanced levels of pro-inflammatory cytokines including IL (interleukin)-1α, IL-1ra (interleukin-1 receptor antagonist), IL-1β, IL-3, IL-5, IL-9, IL-10, IL-12, IL-13, IL-17, IFN (interferon)-γ, eotaxin, TNF (tumor necrosis factor)-β, and MCP (monocyte chemotactic protein)-3 in *APOE ε3/ε4* carriers compared to *ε3/ε3* carriers (Fig. 4b–o). Other cytokines exhibited non-statistically significant differences between *ε3/ε3* and *ε3/ε4* patients (Supplementary Fig. S4a). Together, these results clearly indicate that COVID-19 patients with *ε4* allele exhibit more severe inflammation.

## Discussion

To date, factors determining susceptibility to SARS-CoV-2 remain unclear. In the current study, we identify APOE as a negative regulator for SARS-CoV-2-induced inflammatory response and SARS-CoV-2 pseudo-virus infection. APOE inhibits ACE2-mediated viral Spike protein binding to the cell surface through interaction with ACE2. Interestingly, APOE possesses a motif similar to ghrelin, an ACE2 binding protein and substrate, providing indirect evidence corroborating our findings^24^ (Supplementary Fig. S4b). We also demonstrate that APOE4 inhibits ACE2/Spike interactions and impedes cellular entry of SARS-CoV-2 pseudo-viruses to a lesser extent compared to APOE3. Increased viral load may result in increased inflammation and disease severity, as demonstrated by increased frequency of *ε4* allele in COVID-19 patients and elevated levels of inflammatory factors in the patients with *APOE ε4* genotype. The differential effects of APOE isoforms on ACE2/Spike interactions may be due to conformational differences between APOE3 and APOE4: the N-terminal domain of APOE4 interacts more closely with its C-terminal domain, resulting in a more compact structure of APOE4, when compared to APOE3.^25^ Therefore, it is conceivable that APOE3/ACE2 complexes render larger spatial obstacles compared to APOE4/ACE2 complexes, thereby more efficiently hindering Spike binding to ACE2 (Supplementary Fig. S4c). Interestingly, a recent study shows that the *ε4* carriers (ε3/ε4 or ε4/ε4) have lowest plasma levels of APOE among all APOE genotypes.^26^ Low protein levels and compact structural feature of APOE4 may act synergistically to cause a decreased inhibitory effect on SARS-CoV-2 viral load, and thereby resulting in increased inflammation and disease severity in COVID-19 patients with *APOE ε4* genotype, compared to those with *APOE ε3* genotype. Nevertheless, how APOE regulates ACE2/Spike binding requires further investigations. Particularly, it would be interesting and await to be determined whether key transmembrane proteins such as TMPRSS2 that are involved in cellular entry of SARS-CoV-2 may also play a role in APOE-mediated inhibition of ACE2/Spike interaction.^1^

SARS-CoV-2 infection has recently been implicated to cause brain changes and neurodegeneration. Analyses of multi-modal brain imaging data showed that gray matter thickness in the orbitofrontal cortex and global brain size are reduced in individuals who have been exposed to SARS-CoV-2 infection, when compared to same individuals before the viral infection.^27^ Likewise, APOE4 has also been known to associate with brain structural changes, e.g., hippocampal atrophy and reduced cortical surface area.^28^ A role for APOE4 in mediating SARS-CoV-2 infection of iPSC (induced pluripotent stem cell) -derived neurons and astrocytes was recently reported, where APOE4 enhanced astrocytic response to SARS-CoV-2 infection.^29^ In addition, APOE4 is the most potent risk factor for late-onset AD, and given that recent studies have also shown that AD is associated with higher morbidity and mortality of COVID-19,^30–33^ potential epidemiological correlations between APOE4-mediated AD onset and COVID-19 may be likely. Results from our study suggest that APOE4 is a pivotal mediator that links the susceptibility of COVID-19 to AD. APOE genotype has also been associated with other viral HSV-1 (herpes simplex virus type 1) and HIV-1 (human immunodeficiency virus type 1) infection,^34,35^ which also requires interactions with viral docking proteins.^36^ Future studies may determine whether APOE concentrations and APOE4 status can likewise modulate viral infectivity and severity in other infectious diseases.

In addition to the original report, several recent studies have confirmed the association of APOE4 with severity and mortality of COVID-19 in large cohorts.^37,38^ However, APOE was not identified as a critical host gene for COVID-19 in a recent whole genome sequencing study.^4^ Another study failed to identify a linkage between APOE locus and COVID-19 severity in stratified analyses by dementia status.^39^ The decrepancies in outcomes from different genetic studies may be partillay due to varying criteria in defining COVID-19 severity. Nevertheless, our study supports the notion that APOE4 associates with disease severity, as documented by the increased inflammation in COVID-19 patients with *APOE ε4* genotype, and weaker ability of APOE4 in inhibiting cellular entry of SARS-CoV-2, when compared to the *ε3* carriers and APOE3, respectively. More comprehensive and systemic studies with multiple approaches are needed to further elucidate how APOE is involved in COVID-19.

While APOE4 associates with a higher risk for a number of diseases such as AD, cardiovascular disease and COVID-19, it could exhibit protective benefits under some circumstances. For example, the *ε4* allele is associated with improved fertility and control of infectious diseases under adverse environment in the pre-industrialized era.^40^ Therefore, APOE4 may have an antagonistic pleiotropy, which certainly warrants further scrutiny.

## Materials and methods

### Study design

The purpose of this study was to define how APOE protein and its isoforms affect the SARS-CoV-2 virus entry, and whether and how *APOE ε4* allele may increase susceptibility and severity of COVID-19. Experiments include in vitro and in vivo pseudo-viral entry and protein interaction assessments. Pseudo-viral entry analyses include confocal imaging, flow cytometry analyses and quantitative real-time PCR (qRT-PCR). Protein interaction assays include protein pull-down assay, Bio-Layer-Interferometry assay, live-cell surface staining, stimulated emission depletion microcopy (STED), and immuno-electron microscopy. *APOE* allele genotyping was carried out by Sanger-sequencing and qRT-PCR, and Cytokine/chemokine assays were conducted using multiplex ELISA. WT, APOE3-TR and APOE4-TR mice were used for intranasal infection of SARS-CoV-2 pseudo-virus. The rate of infection was analyzed by confocal imaging and qRT-PCR. The investigators were blinded to APOE genotypes. The number of experimental or biological replicates is specified in the figure legends.

### Mice

C57BL/6J wild-type (WT) mice were from the Laboratory Animal Center at Xiamen University. Human APOE3-targeted replacement (APOE3-TR) (B6. Cg-Apoeem2(APOE*) Adiuj/J) and APOE4-TR (B6(SJL)-Apoetm1.1(APOE*4) Adiuj/J) mice were from Jackson Laboratory. All animals were maintained and animal experiments were performed following instructions from the Institutional Animal Care and Use Committee of Xiamen University and Chongqing Medical University.

### Cell culture and transfection

293T cells (Cat#CRL-3216) were purchased from ATCC, BEAS-2B(Cat#C6106) cells from Beyotime, 293T-ACE2 (Cat#HEK-293T-LV-0582) cells were purchased from BrainVTA, and were cultured in Dulbecco’s Modified Eagle Medium (DMEM) containing 10% fetal bovine serum (FBS) and 1% penicillin/streptomycin (P/S). Calu-3 (Cat#CL-0054) cells were purchased from Procell, and cultured in Modified Eagle Medium (MEM) supplemented with 10% FBS and 1% P/S. Murine astrocytes overexpressing hAPOE3 or hAPOE4 were a kind gift from David Holtzman (Washington University), and maintained in DMEM with 20% FBS and 1% P/S. All cells were tested negative for *Mycoplasma* spp. Various constructs encoding Flag- or HA- tagged APOE, HA- and His-tagged full-length or fragmented ACE2, were transfected into 293T or 293T-ACE2 cells with polyethyleneimine (PEI). Cells were subjected to various analyses 24 h after the transfection.

### Mouse challenge experiments

Eight-week-old WT, APOE3-TR, and APOE4-TR mice were anesthetized, and then intranasally administrated with AAV-ACE2-His (BrainVTA, Cat#PT-2765) (1 × 10^7^ PFU/mouse). Mice were monitored daily and intranasally transduced with VSV-ΔG-SARS-CoV-2-EGFP (BrainVTA, Cat#V04001) (1 × 10^6^ PFU/mouse) on day 7 post AAV transduction. Mice were sacrificed 14 days after AAV transduction and lung tissues were dissected and processed for immunofluorescence staining. Briefly, tissues were fixed in 4% paraformaldehyde (PFA) in PBS for 8 h followed by cryoprotection in 20% sucrose for 8 h, and then in 30% sucrose overnight. Tissues were frozen in optimal cutting temperature compound (Sakura, Cat #4583) and sectioned at 15 microns. Slides were rehydrated in PBS for 10 min, stained with DAPI, mounted in Vectashield antifade mounting medium (Vector Laboratories, Cat#H-1000-10), and subjected to confocal imaging on an LSM 880 confocal microscope (Zeiss).

### RNA extraction and qRT-PCR

Lung tissues were dissected from mice transduced with AAV-ACE2-His and VSV-ΔG-SARS-CoV-2-EGFP and immersed in liquid nitrogen and stored at –80 °C. RNA was extracted from individual tissue using TRIzol (Invitrogen, Cat#15596-026) and reverse transcribed to cDNA using FastQuant RT kit (TIANGEN, Cat#KR106). qRT-PCR was carried out on the Light Cycler 480 System (Roche) using the FastStart Universal SYBR Green Master (Roche, Cat#04913850001) and primers targeting specific genes, including human *ACE2 (F: CTTTGAGCCCTTATTTACCTGGCTG, R: TACATTTCATTGTCGTTCCATTCATATGC), EGFP (F: GCCACAAGTTCAGCGTGTCC, R: GGGTAGCGGCTGAAGCACTG)*, and mouse *Gapdh (F: CATCACTGCCACCCAGAAGACTG, R: ATGCCAGTGAGCTTCCCGTTCAG)*. Relative

expression was determined using the comparative Ct model (ΔΔCt) with glyceraldehyde 3-phosphate dehydrogenase (Gapdh) as an internal control.

### Pseudo-viral entry assay

293T-ACE2 or 293T cells (1 × 10^5^ cells/well) were seeded into 24-well plates 24 h before treatment. To simulate physiological conditions, cells were incubated with different concentrations of recombinant human APOE3 or APOE4 protein (0, 2, 8 µg/mL) for 2 h before viral infection. Cells were then transduced with pseudo-viruses (2 × 10^4^ IFU/well) for an additional 24 h, and washed and fixed for confocal imaging (Zeiss, LSM 880) or flow cytometry (Beckman, CytoFlex S) analyses. The percentage of EGFP-positive cells was calculated and analyzed. Alternatively, 293T-ACE2 or 293T cells were transduced with the pseudo-viruses for 24 h, and viruses were then removed and recombinant human APOE3 or APOE4 (8 µg/mL) was included into the culture medium for 2 h (data obtained following this procedure were specified in the Figure Legends). Cells were washed and fixed for confocal imaging or flow cytometry analyses.

### Protein pull-down assay

Protein pull-down assays were conducted as described previously.^41^ Briefly, experimental cells were lysed with 200 μL TNEN (20 mM Tris-HCl, 100 mM NaCl, 1 mM EDTA, 0.5% NP40, pH 7.4) buffer for 15 min at 4 °C, and then centrifuged at 12,000 × *g*, 4 °C for 10 min. 0.25 mg Protein G magnetic beads (Invitrogen, Cat#10007D) were incubated with 2 μg anti-ACE2 antibody (ABclonal, Cat#A4612) or normal rabbit control IgG (SinoBiological, Cat#CR1) on a rotator. Beads were washed three times with PBS containing 0.05% Tween-20 (PBST) and incubated with the cell lysates for 30 min at room temperature (RT). Alternatively, 0.25 mg anti-Flag Magnetic Beads (MCE, Cat#HY-K0207) were washed with PBST and added to the cell lysates for 30 min at RT. After PBST washing, proteins bound to beads were eluted with a buffer containing 50 mM sodium citrate (pH 3.5) and boiled in sample buffer containing 2% SDS.

For three-protein pull-down assays: 0.25 mg Protein G crosslinked-Dynabeads were firstly incubated with 2 μg ACE2-Fc (SinoBiological, Cat#10108-H02H) or Fc (SinoBiological, Cat#10702-HNAH) protein, washed with PBST, and then incubated with 0.4 μg APOE3 or APOE4 for 30 min at RT. After washing with PBST, bound proteins were subjected to elution and boiling.

Boiled samples were subjected to Western blot analyses and detected using antibodies against ACE2 (ABclonal, Cat#A4612), Flag (ProteinTech, Cat# 66008-4-Ig), human Fc (ProteinTech, Cat#660051) or APOE (Meridian Life Science, Cat#K74180B).

### Bio-layer-interferometry (BLI) assay

BLI assays were performed using the Octet RED96 system (ForteBio). ACE2-His fusion proteins (10 μg/mL) were captured on Ni-NTA Biosensors (ForteBio, Cat#18-5101) and incubated with APOE3 or APOE4 protein at various concentrations (62.5 nM, 125 nM, 250 nM, 500 nM, 1000 nM). The experiments include four steps: (i) ACE2-His (SinoBiological, Cat#10108-H08H) protein loading onto Ni-NTA Biosensors (300 s); (ii) baseline; (iii) association of APOE3 or APOE4 for determining *K*_on_ (120 s); and (iv) dissociation of APOE3 or APOE4 for *K*_dis_ measurement (600 s). Baseline and dissociation steps were performed in SD buffer (pH 7.4 PBS,0.05% tween20, 0.01% BSA) and biosensor drifting was corrected by background subtraction. Background wavelength shifts were measured from reference biosensors that were only loaded with ACE2-His in SD buffer. All steps were conducted with shaking at 1000 rpm, 30 °C. The data were analyzed and fitted in a 1:1 binding model by Octet data analysis software (ForteBio, version 9.0).

### Immuno-electron microscopy

Cells were fixed in 0.1 M sodium cacodylate buffer (pH 7.4) containing 0.5% glutaraldehyde and 2% PFA for 4 h at 4 °C, and then rinsed in 0.01 M sodium cacodylate buffer at pH 7.4. Cells were dehydrated with a gradient series (30%, 50%, 70%, 90% and 100%) of ethanol, and infiltrated with LR White resin (London Resin). Samples were embedded into gelatin capsules, polymerized under UV light for 48 h at 4 °C, sectioned at 90 nm thickness and loaded onto a non-coated nickel grid (size 300 mesh). The dried sections were blocked with Tris-buffered saline (pH 7.4) containing 1% BSA for 10 min at RT, and incubated with the following antibodies: rabbit anti-ACE2 (Abclonal, Cat#A4612), mouse anti-SARS-CoV-2 spike glycoprotein RBD (abcam, Cat#ab277628), or mouse anti-APOE (Novus Biologicals, Cat#NB110-60531) at 4 °C for 12 h. Samples were then incubated with 18 nm colloidal gold AffiniPure donkey anti-rabbit IgG (Jackson ImmunoResearch, Cat#711-215-152) and 12 nm colloidal gold AffiniPure donkey anti-mouse IgG (Jackson ImmunoResearch, Cat#715-205-150) for 3 h at RT. Finally, samples were contrasted by a 10-minitue incubation with 2% aqueous uranyl acetate and imaged on an HT-7800 transmission electron microscope system (Hitachi).

### Live-cell surface staining and STED

293T-ACE2 cells were incubated with Spike-Fc (SinoBiological, Cat#40591-V02H), Spike-Fc/APOE3, and Spike-Fc/APOE4 in culture medium for 1 h at 4 °C, respectively. Cells were washed three times in PBS, and then fixed in 4% PFA in PBS for 10 min at RT. Fixed samples were rinsed in PBS,blocked in PBS containing 10% donkey serum for 1 h at RT, and incubated with antibodies against ACE2 or SARS-CoV-2 spike glycoprotein RBD for 12 h at 4 °C. After washing with PBS, cells were incubated with secondary antibodies: star red-conjugated goat anti-mouse (Abberior Instruments, Cat#NC1933868) or star orange-conjugated goat anti-rabbit (Abberior Instruments, Cat#NC1933866) for 1 h at RT. Cell nuclei were labeled with DAPI for 20 min at RT. After PBS washing, samples were mounted in Vectashield mounting medium and imaged with a STEDYCON (Abberior Instruments) on an inverted Nikon microscope.

### Ethical approval

Use of human blood samples was approved by the Ethics Commission of Chongqing Medical University (Reference# 2020004). Written informed consent for participation was obtained from all adult participants or guardians on behalf of the children enrolled in this study.

### Cytokine/chemokine measurement

Serum from patients with laboratory-confirmed SARS-CoV-2 infections were collected as early as possible during hospitalization. Levels of 48 cytokines and chemokines in serum samples were determined as previously described.^42^ Briefly, 10 μL serum samples from each individual were loaded into a Bio-Plex Human Cytokine Screening Panel (Bio-Rad, Cat#12007283) and analyzed on a Luminex 200 Instrument System (Merck Millipore) following the manufacturer’s instructions.

### APOE genotyping

Genomic DNA was purified from blood samples using a kit from Qiagen (Cat# 69504). APOE SNPs (rs429358: T/C and rs7412: C/T) were detected using APOE genotyping kits (Memorigen Biotech, Cat# 20173403322) and a LightCycler 96 Instrument (Roche). APOE genotypes were determined by the allele-specific fluorescence.^43^ The analyses were repeated at least twice, and blinded between different investigators. All genotypes were confirmed by Sanger sequencing.

### APOE protein measurement

Serum samples from COVID-19 patients were diluted 10 times with horse serum (Kangyuan, Tianjin, China) and analyzed on the AST-Sc-Lite (A fully-auto single-molecule detection machine supplied by Suzhou AstraBio technology Co., Ltd.) according to manufacturer’s instructions. Briefly, the working steps include:(i) Load 25 μL sample into an incubation tube and add Reagent 1 (mainly comprised 0.1 mg/mL magnetic beads coated with capture antibodies and protecting reagents), followed by a quick mixing by the machine. (ii) After a 3-min incubation period, Reagent 2 (mainly comprised APOE detection antibodies labeled with single-molecule imagine fluorophores supplied by AstraBio) was added, mixed and incubated for 2 min under 40 °C. (iii) Magnetic beads in the mixtures were absorbed onto the surface of the channel in the flow cell by a permanent magnet. Unlabeled fluorophores were removed by a gentle washing flow of wash buffer and fluorescent images were then taken with an integrated fluorescent microscope. (IV) The single-molecule signals were analyzed by the machine and protein concentrations were calculated with a standard curve prepared in advance.

### Statistical analysis

All experiments were repeated at least three times. All data are presented as mean ± S.E.M. Unpaired, two-sided Mann–Whitney *U*-test, one-way or two-way ANOVA tests were used to determine statistical significance. *P < 0.05; **P < 0.01; ***P < 0.001, ****P < 0.0001.

## Supplementary information

Supplementary

## Data Availability

This study did not generate any unique datasets or code.
