# Supplementary material for: APOE interacts with ACE2 inhibiting SARS-CoV-2 cellular entry and inflammation in COVID-19 patients
Source: Signal Transduct Target Ther. 2022 Aug 1;7:261. doi: 10.1038/s41392-022-01118-4 (PMC9340718; doi:10.1038/s41392-022-01118-4)
Supplement: Supplementary file 1 — Supplementary [file 41392_2022_1118_MOESM1_ESM.docx]

Supplementary Materials for

**APOE interacts with ACE2 inhibiting SARS-CoV-2 cellular entry and inflammation in COVID-19 patients**

Hongsheng Zhang,^1,2†^ Lin Shao,^3†^ Zhihao Lin,^3†^ Quan-Xin Long,^4†^ Huilong Yuan,^3†^ Lujian Cai,^3^ Guangtong Jiang,^3^ Xiaoyi Guo,^3^ Renzhi Yang,^2^ Zepeng Zhang,^1^ Bingchang Zhang,^1^ Fan Liu,^1^ Zhiyong Li,^1^ Qilin Ma,^1^ Yun-Wu Zhang,^1,3^ Ai-Long Huang,^4*^ Zhanxiang Wang,^1*^ Yingjun Zhao^1,3*^ and Huaxi Xu^1,2*^

Correspondence to: hxxu@xmu.edu.cn

**This PDF includes:**

Figures.S1 to S4

Tables S1 to S2


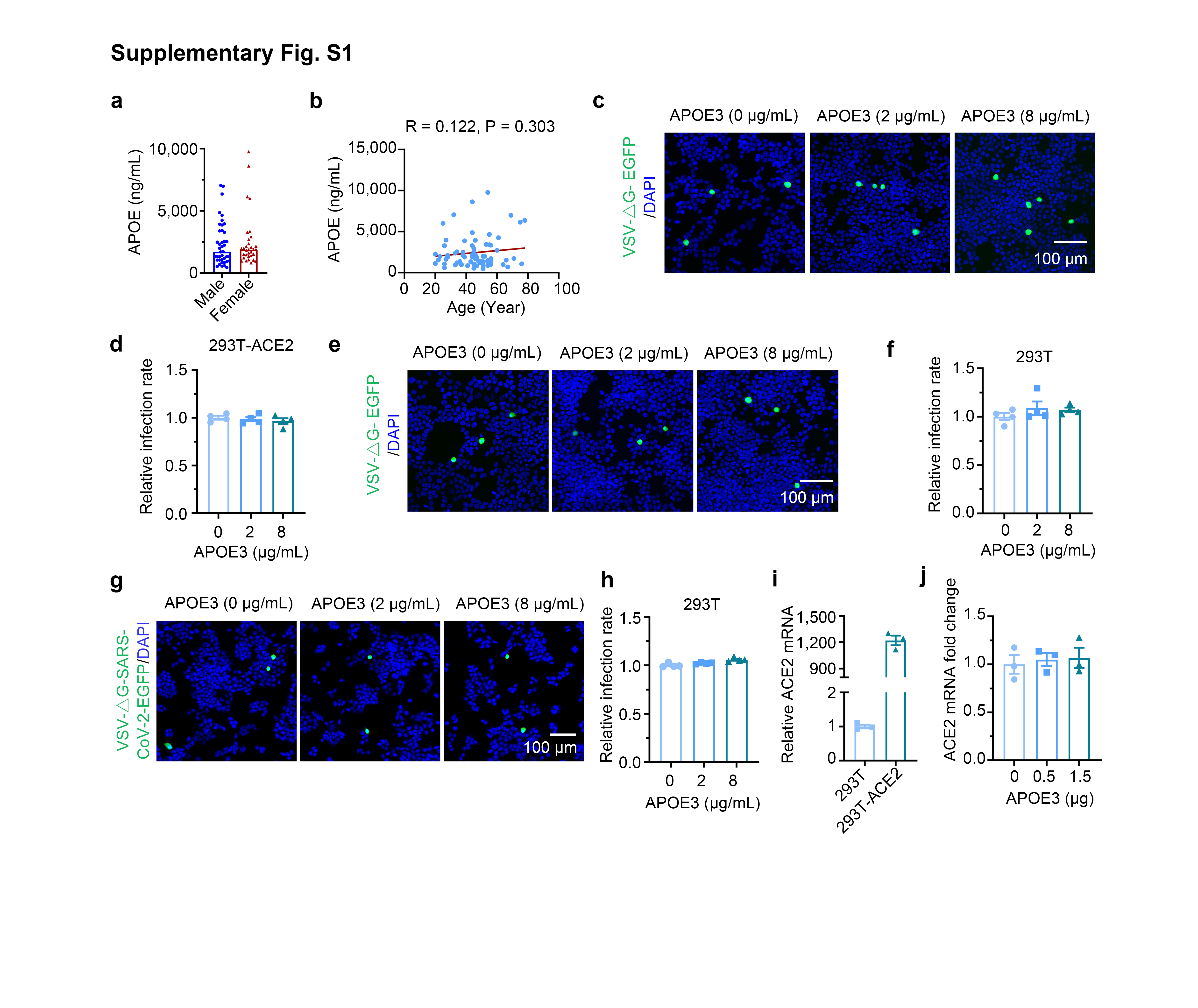


**Figure. S1. Correlation analysis between sex, age and APOE concentrations in serum samples from COVID-19 patients and the effect of APOE3 on viral transduction.**

**a** Analysis of serum APOE concentrations in male and female COVID-19 patients. n = 41 for males, n = 32 for females. **b** Analysis of correlation between APOE concentrations and age. n = 73. **c-f** Confocal imaging analysis of VSV-△G-EGFP viral load in 293T-ACE2 cells (**c, d**) or 293T cells (**e, f**) treated with varying concentrations of recombinant APOE3 proteins. n = 4 independent experiments. **g-h** Confocal imaging analysis of VSV-△G-SARS-CoV-2-EGFP viral load in 293T cells treated with varying concentrations of recombinant APOE3 proteins. n = 4 independent experiments. **i** qRT-PCR analysis of ACE2 mRNA levels in 293T and 293T ACE2 cells. **j** qRT-PCR analysis of human ACE2 mRNA levels in mouse lung tissues transduced with AAV-ACE2-His and treated with recombinant APOE3 proteins. n = 3 mice per group. Data are presented as mean ± S.E.M. One-way ANOVA tests were used to determine statistical significance.


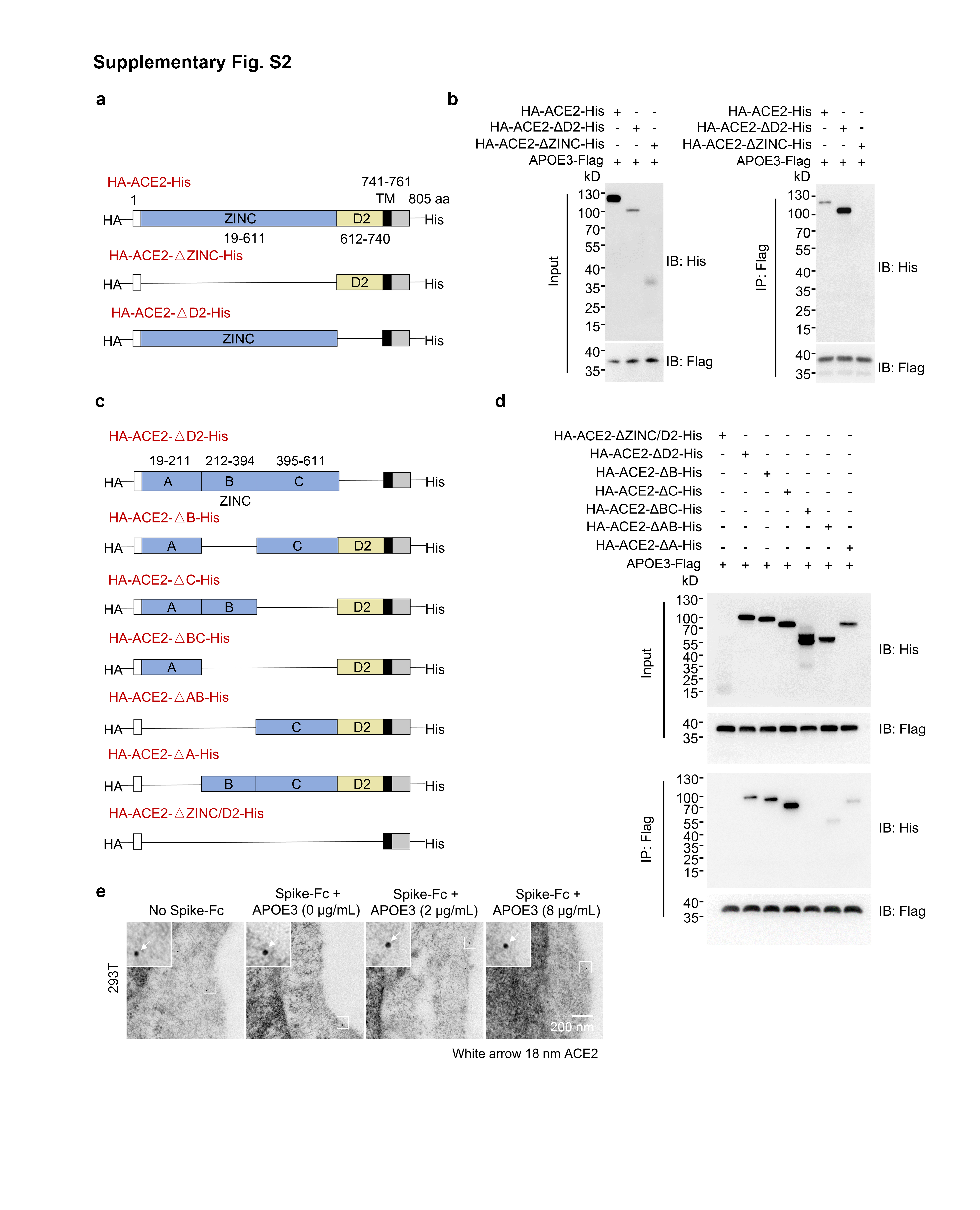


**Figure. S2. APOE3 interacts with B and C regions of the ACE2 zinc domain.**

**a-d** Co-immunoprecipitation analysis of interactions between tagged APOE3 and ACE2 fragments or full-length protein in 293T cells. **a, c** Schematic of constructs used for Co-IP. TM: transmembrane domain. **b, d** Representative images of immunoblots. **e** Immuno-electron microscopic analysis of the amount of Spike-Fc protein bound to the surface of 293T cells in the presence of recombinant APOE3 proteins at varying concentrations. n = 4 independent experiments.


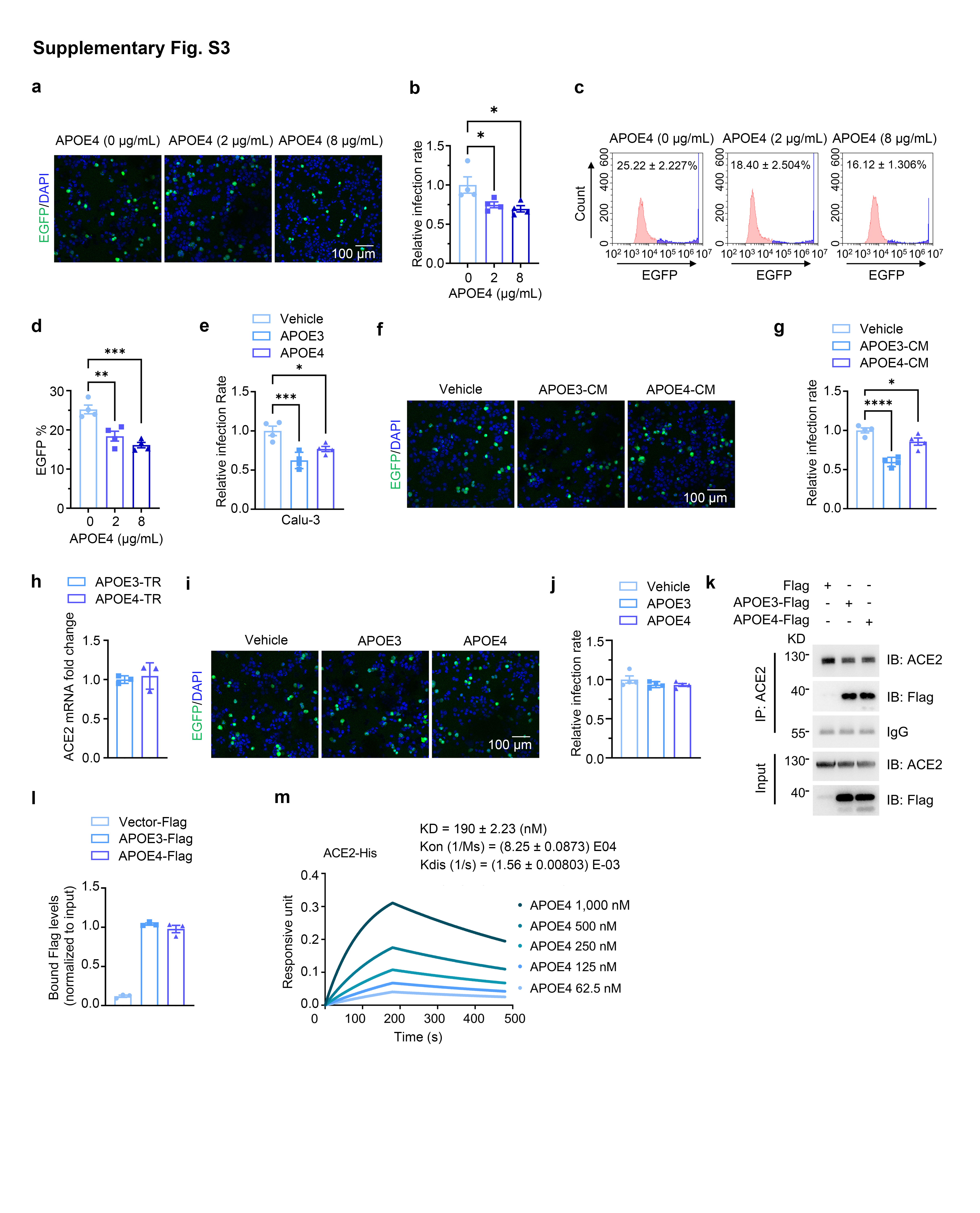


**Figure. S3. APOE4 also dose-dependently inhibits cellular entry of SARS-CoV-2 pseudo-virus but to a lesser extent compared to APOE3.**

**a-d** Confocal imaging **(a-b)** and flow cytometry **(c-d)** analyses of VSV-△G-SARS-CoV-2-EGFP pseudo-viral load in 293T-ACE2 cells treated with varying concentrations of recombinant APOE4 proteins. n = 4 independent experiments. **e** Analysis of VSV-△G-SARS-CoV-2-EGFP pseudo-viral load in Calu-3 cells treated with recombinant APOE3 or APOE4 proteins. n = 4 independent experiments. **f-g** Confocal imaging analysis of VSV-△G-SARS-CoV-2-EGFP pseudo-viral load in 293T-ACE2 cells treated with conditioned medium (CM) from APOE3-TR or APOE4-TR mouse astrocytic cultures. CM was added 2 hours before viral transduction and cellular EGFP positivity was evaluated 24 hours after viral infection. n = 4 independent experiments. **h** qRT-PCR analysis of human ACE2 mRNA levels in APOE3-TR or APOE4-TR mouse lung tissues transduced with AAV-ACE2. n = 3 mice per group. **i-j** Confocal imaging analyses of VSV-△G-SARS-CoV-2-EGFP pseudo-viral load in 293T-ACE2 cells. Cells were transduced with the pseudo-viruses for 24 hours and then removed and incubated with recombinant APOE3 or APOE4 proteins for 2 hours. n = 4 independent experiments. **k-l** Co-immunoprecipitation between APOE3/APOE4 and ACE2 in 293T-ACE2 cells overexpressing APOE3/4-Flag. **m** Bio-layer interferometry analysis of immobilized ACE2-Fc proteins bound to APOE4 proteins at the following concentrations: 1000 nM, 500 nM, 250 nM, 125 nM, and 62.5 nM (corresponding to kinetic curves from top to bottom). Data are presented as mean ± S.E.M. Unpaired, two-sided Mann-Whitney U test was used to determine statistical significance. *, *P* < 0.05; **, *P* < 0.01; ***, *P* < 0.001; ****, *P* < 0.0001.


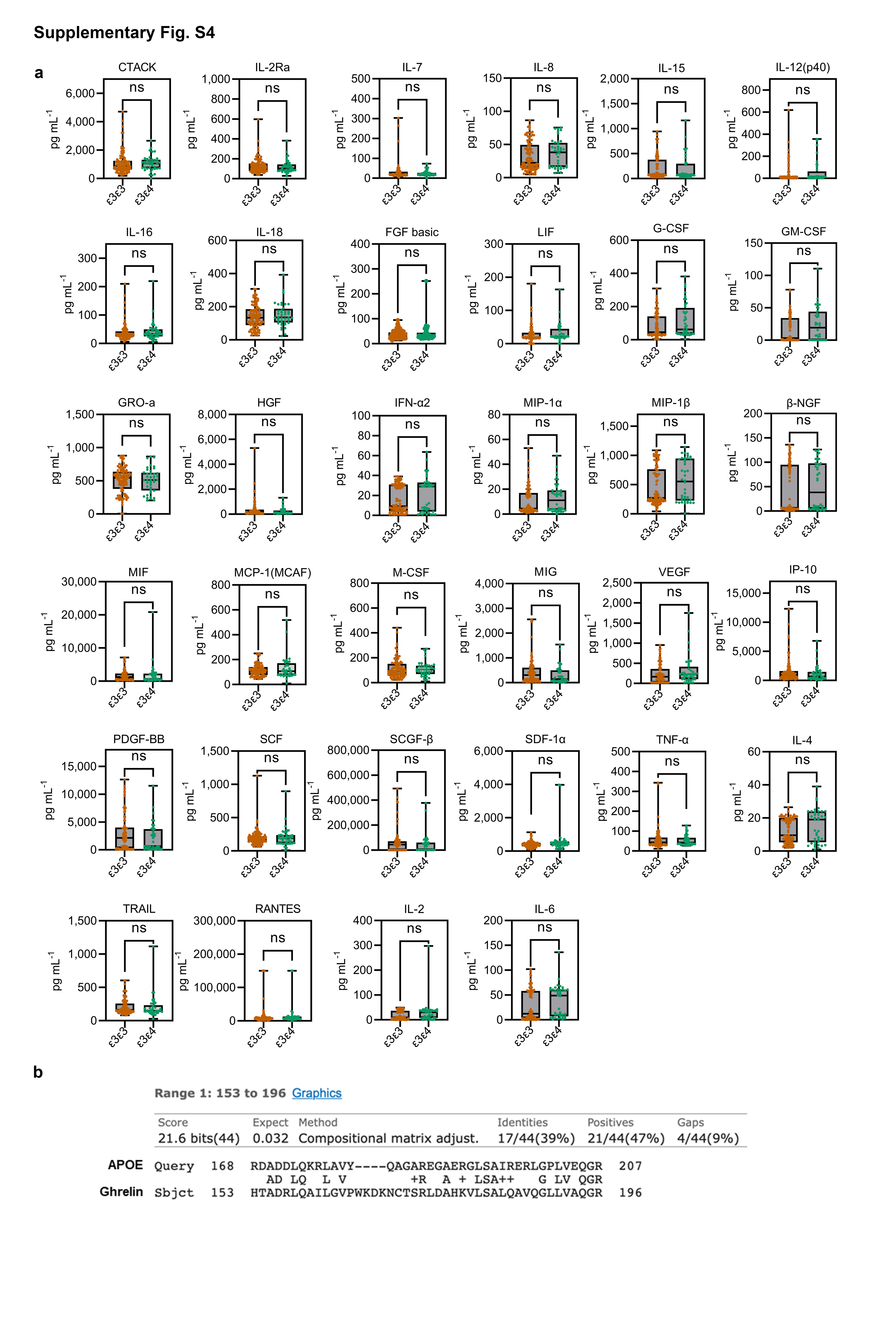

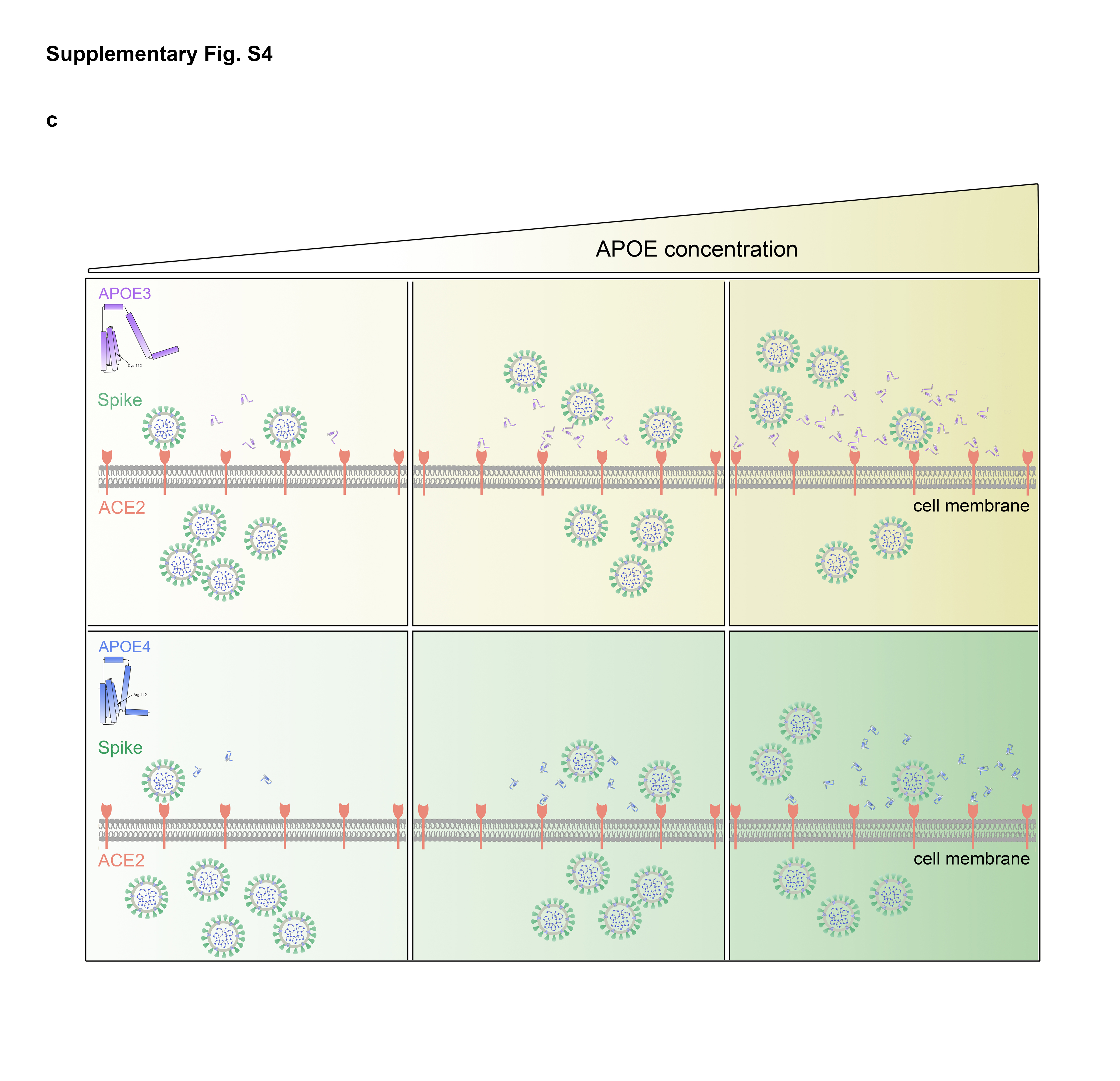


**Figure. S4. Concentration of inflammatory factors in serum samples from COVID-19 patients with APOE ε3/ε3 (n = 102) or ε3/ε4 (n = 40) genotypes, alignment of APOE and Ghrelin partial protein sequences, and a schematic model for APOE-mediated regulation of SARS-CoV-2 infection.**

**a** Serum concentration of cytokines and chemokines were measured by ELISA. Box plots represent median, first and third quartiles; Whiskers represent 1.5x the IQR (interquartile range) above and below the box. Data are presented as mean ± S.E.M. Unpaired, two-sided Mann-Whitney U test was used to determine statistical significance. ns: no significance. **b** Comparison of homology between APOE and Ghrelin, an ACE2 binding protein and substrate (generated from https://blast.ncbi.nlm.nih.gov/blast.cgi). **c** APOE dose-dependently inhibits ACE2/Spike-mediated entry of SARS-CoV-2 into cells. APOE4 shows less inhibitory effect on SARS-CoV-2 infection because the structure of APOE4 is more compact than of APOE3, resulting in a smaller spatial obstacle for the binding of Spike to ACE2.

**Table S1. Information for human serum samples used for APOE concentration examination.**

| Group | Sex | n | Age (years) |
| --- | --- | --- | --- |
| APOE ε3/ε3 | Male | 41 | 47.07 ± 2.01 |
| APOE ε3/ε3 | Female | 32 | 44.44 ± 2.60 |

**Table S2. Information for human serum samples used for cytokine analyses in COVID-19 patients with different *APOE* genotypes.**

| Group | Sex | n | Age (years) |
| --- | --- | --- | --- |
| APOE ε3 carrier | Male | 52 | 46.93 ± 1.81 |
| APOE ε3 carrier | Female | 50 |  |
| APOE ε4 carrier | Male | 24 | 49.20 ± 2.45 |
| APOE ε4 carrier | Female | 16 |  |
| t，df |  |  | t = 0.694, df = 140 |
| P |  |  | 0.489 |
